# SUMO E3 ligase SIZ1 negatively regulates arsenite resistance via depressing GSH biosynthesis in Arabidopsis

**DOI:** 10.1007/s44154-021-00029-8

**Published:** 2022-01-27

**Authors:** Yechun Hong, Yunjuan Chen, Huazhong Shi, Xiangfeng Kong, Juanjuan Yao, Mingguang Lei, Jian-Kang Zhu, Zhen Wang

**Affiliations:** 1 Shanghai Center for Plant Stress Biology and Center for Excellence in Molecular Plant Sciences, Chinese Academy of Sciences, Shanghai, 200032 China; 2 University of Chinese Academy of Sciences, Beijing, People’s Republic of China; 3 Department of Chemistry and Biochemistry, Texas Tech University, Lubbock, TX 79409 USA; 4 School of Life Sciences, Anhui Agricultural University, Hefei, 230036 China

**Keywords:** Arsenite, GSH, PHR1, SIZ1, SUMOylation

## Abstract

Arsenic is a metalloid toxic to plants, animals and human beings. Small ubiquitin-like modifier (SUMO) conjugation is involved in many biological processes in plants. However, the role of SUMOylation in regulating plant arsenic response is still unclear. In this study, we found that dysfunction of SUMO E3 ligase SIZ1 improves arsenite resistance in Arabidopsis. Overexpression of the dominant-negative SUMO E2 variant resembled the arsenite-resistant phenotype of *siz1* mutant, indicating that SUMOylation plays a negative role in plant arsenite detoxification. The *siz1* mutant accumulated more glutathione (GSH) than the wild type under arsenite stress, and the arsenite-resistant phenotype of *siz1* was depressed by inhibiting GSH biosynthesis. The transcript levels of the genes in the GSH biosynthetic pathway were increased in the *siz1* mutant comparing with the wild type in response to arsenite treatment. Taken together, our findings revealed a novel function of SIZ1 in modulating plant arsenite response through regulating the GSH-dependent detoxification.

Rapid and dynamic SUMO conjugation of cellular proteins is known to be crucial in plant adaption to environmental changes (Morrell and Sadanandom, 2019). In Arabidopsis, the SUMO E3 ligase SIZ1 is essential for SUMOylation of the substrates mediating stress responses (Augustine and Vierstra, 2018). To investigate whether the SIZ1-mediated SUMOylation is involved in plant response to arsenic toxicity, we first performed a phenotypic assay of the Arabidopsis T-DNA insertion mutants *siz1–2* (SALK_065397) and *siz1–3* (SALK_034008) in response to arsenite treatments. Under normal growth conditions without arsenite, the *siz1* mutant plants had reduced fresh weight but similar root length comparing with the wild type. However, when treated with 25 μM arsenite, the mutant plants showed increased fresh weight and root elongation when compared with the wild type (Fig. 1a-c). These results indicated that SIZ1 negatively regulates arsenite resistance in Arabidopsis. Since natural inorganic arsenic compounds that can be absorbed by and toxic to plants mainly consist of trivalent arsenite [As (III)] and oxidative pentavalent arsenate [As(V)] (Ashraf et al., 2020), we also tested the response of *siz1* mutants to arsenate stress. The results showed that the *siz1* mutants and Col-0 wild type responded similarly to arsenate treatment (Fig. 1d and e), indicating that SIZ1 plays an important role in the detoxification of arsenite but not arsenate in Arabidopsis. We further detected the SUMOylation profiles in Col-0 wild type and *siz1–2* mutant seedlings with or without sodium arsenite treatment. The immunoblot assay using anti-AtSUMO1 antibody showed dynamic changes of SUMO-conjugated products in Col-0 wild type with an increase in SUMOylation after As (III) treatment for 2 h and a decline after longer time treatments, while this pattern was clearly altered in the *siz1–2* mutant (Fig. 1f), which further supports that SIZ1-mediated SUMO conjugation is involved in arsenite response in Arabidopsis.

**Fig. 1:**
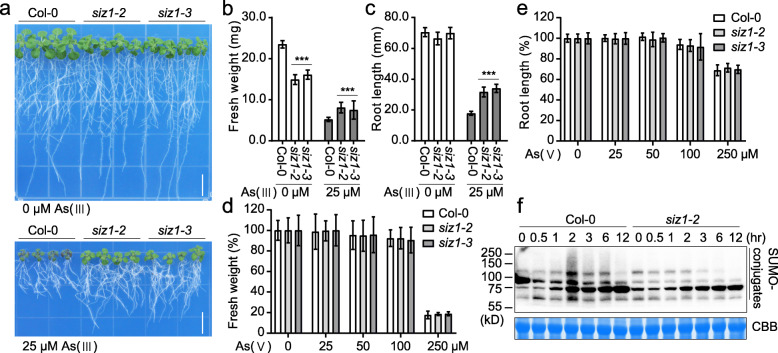
SIZ1 is a negative regulator of arsenite resistance in Arabidopsis. **a** Arsenite resistance assay of Col-0, *siz1–2*, and *siz1–3* seedlings. Five-day-old seedlings grown in 0.5× MS medium were transferred to medium with or without 25 μM As (III) for additional 12 days. Bar, 1 cm. **b**, **c** Fresh weight (**b**) and root length (**c**) of Col-0, *siz1–2*, and *siz1–3* seedlings shown in (**a**). **d**, **e** Fresh weight (**d**) and root length (**e**) of Col-0, *siz1–2*, and *siz1–3* seedlings treated with 0, 25, 50, 100 or 250 μM As(V). Five-day-old seedlings grown in 0.5× MS medium were transferred to medium with or without As(V) for additional 12 days. Values are means ± SD of three replicates, each replicate containing 9 plants per genotype. *** *P* < 0.001, Student’s *t* test. **f** SUMOylation profiles of wild type Col-0 and *siz1–2* seedlings after As (III) treatment. Ten-day-old Col-0 and *siz1–2* seedlings grown in 0.5× MS medium were treated with 100 μM As (III) for the indicated time periods. The SUMOylation profiles were determined by Western blot using anti-SUMO1 antibody. The Coomassie blue (CCB) staining was used as a loading control

The role of SIZ1 in arsenite response was further consolidated by the molecular complementation of the *siz1–2* mutant. The *SIZ1* gene with its native promoter and coding sequence was amplified and cloned into pCambia1305 vector to generate the *SIZ1pro:3×HA-SIZ1* construct, which was then introduced into *siz1–2* mutant. Two independent transgenic lines, which fully rescued the dwarf-like phenotype of *siz1–2* under normal conditions, were designated as Com-1 and Com-2 and used for further analysis. Quantitative reverse transcription (qRT)-PCR analysis showed that the *SIZ1* transcript level was recovered in the complementation lines (Fig. 2a). The immunoblot assay using anti-HA antibody confirmed the expression of the 3×HA-SIZ1 fused proteins in these two complementation lines (Fig. 2b). Phenotypic analysis indicated that the expression of the native *SIZ1* gene rescued the arsenite response phenotype of the *siz1–2* mutant (Fig. 2c and d), revealing that the arsenite-resistant phenotype of *siz1–2* mutant is resulted from the loss of function of *SIZ1* gene. Moreover, we tested the arsenite response of the overexpression line of SUMO E2, designed as SCE1^WT^, and the dominant-negative line of SCE1, named SCE1^C94S^, which was reported in several previous studies (Tomanov et al., 2013). Two independent SCE1^WT^ lines (SCE1^WT^-1 and SCE1^WT^-2), two independent SCE1^C94S^ lines (SCE1 ^C94S^-1 and SCE1 ^C94S^-2), *siz1–2*, and Col-0 wild type were used in the analysis. Under normal growth conditions, the SCE1^C94S^ lines showed a dwarf-like phenotype that resembled the phenotype of *siz1–2* mutant, while the phenotype of SCE1^WT^ lines is similar with the Col-0 wild type. Interestingly, the SCE1^C94S^ plants also displayed arsenite resistant phenotype with increased fresh weight and root length, which was similar to the *siz1–2* mutant, while the SCE1^WT^ plants were comparable to the Col-0 wild type under As (III) stress condition (Fig. 2e and f). The similar arsenite resistant phenotype between the *siz1* mutant and the dominant-negative SCE1 plants manifests the role of SUMOylation in arsenite response in Arabidopsis.

**Fig. 2:**
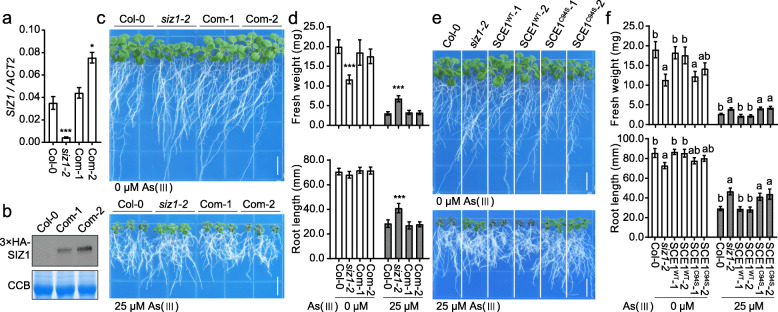
Molecular complementation and mimicking *siz1–2* arsenite-resistance phenotype by dominant-negative SCE1 overexpression. **a** Transcript levels of *SIZ1* determined by qRT-PCR in 18-day-old Col-0, *siz1–2*, Com-1, and Com-2 seedlings. Com-1 and Com-2 are two independent complementation lines of *siz1–2* plants expressing the *SIZ1pro:3*×*HA-SIZ1* transgene. *ACT2* was used as an internal control. **b** Immunoblot analysis of 3×HA-SIZ1 in Col-0, Com-1, and Com-2 plants. The Coomassie blue (CCB) staining was used as a loading control. **c** Arsenite resistance assay of Col-0, *siz1–2*, Com-1, and Com-2 plants. Five-day-old seedlings grown on 0.5× MS medium were transferred to medium with or without 25 μM As (III), and the photograph were taken after treatment for 12 days. Bar, 1 cm. **d** Fresh weight and root length of Col-0, *siz1–2*, Com-1, and Com-2 shown in (**c**). Values are means ± SD of three replicates, each replicate containing 9 plants per genotype. *** *P* < 0.001, Student’s *t* test. **e** Arsenite sensitivity assay of Col-0, *siz1–2* and transgenic lines expressing the E2 conjugation enzyme SCE1. Five-day-old seedlings grown on 0.5× MS medium were transferred to the medium containing 0 or 25 μM As (III) and treated for 12 days. SCE1^WT^-1 and SCE1^WT^-2, two independent lines of *35S:SCE1*^*WT*^*-3×FLAG* in Col-0 plants. SCE1^C94S^-1 and SCE1^C94S^-2, two independent lines of *35S:SCE1*^*C94S*^*-3×FLAG* in Col-0 plants. Bar, 1 cm. **f** Fresh weight and root length measured from the seedlings shown in (**e**). Values are means ± SD of three replicates, each replicate containing 9 plants per genotype. The letters a and b above columns indicate significant difference relative to Col-0 and *siz1–2* mutant, respectively (*P* < 0.01, Student’s *t*  test)

The uptake of pentavalent arsenate is mediated by the phosphate transporters and the cellular arsenate is then converted into trivalent arsenite by the function of arsenate reductases (LeBlanc et al., 2013; Chao et al., 2014). The cytotoxic arsenite is either extruded from the cytoplasm or complexed with thiol(−SH)-rich peptides, and the formation of arsenite-SH is conducive to reducing the translocation of the harmful arsenite in plants (Tripathi et al., 2007). To explore the molecular mechanism of arsenite-resistance conferred by the *siz1* mutations, we measured the contents of arsenic in shoots and roots of *siz1–2*, *siz1–3* and Col-0 wild type seedlings after As (III) treatment to determine whether SIZ1 controls arsenite uptake and accumulation. The result showed no significant differences in arsenic contents in shoot or root of *siz1* mutants and Col-0 wild type seedlings (Fig. 3a and b), suggesting that *siz1* mutations did not affect the uptake and accumulation of arsenite. Heavy metals and metalloids lead to excessive production of reactive oxygen species (ROS) which is detoxified by reductive glutathione (GSH) in plants (Yadav, 2010). In addition, glutathione results in the synthesis of thiol(−SH)-rich metal-binding peptides, the phytochelatins (PCs) that are involved in heavy metal tolerance (Angulo-Bejarano et al., 2021). To determine whether these detoxification mechanisms are involved in arsenite tolerance of *siz1* mutant, we measured the contents of GSH and its precursor cysteine in *siz1* mutants and Col-0 wild type seedlings under normal and As (III) treatment conditions. The *siz1* mutants had significantly higher GSH contents than the Col-0 wild type plants under both normal and As (III) treatment conditions, whereas the contents of cysteine were significantly lower in *siz1* mutants than Col-0 wild type (Fig. 3c and d). To further evidence the contribution of GSH accumulation to the As (III) tolerance of *siz1* mutants, we tested the As (III) sensitivity of *siz1* mutants in the presence of the GSH biosynthesis inhibitor L-buthionine sulfoximine (L-BSO) (Schnaubelt et al., 2015). When supplemented with 100 μM L-BSO, seedling growth was not affected under normal conditions, while the arsenite resistance phenotype of *siz1* mutants completely disappeared in the medium with 25 μM As (III) (Fig. 3e and f). These results indicate that increased GSH biosynthetic accumulation is responsible for the arsenite resistance of *siz1* mutants. We therefore determined the transcript levels of the key genes involved in GSH biosynthesis and metabolism. The results showed that the expression of *GSH1*, *GSH2*, *PCS1* and *PCS2* were increased in *siz1–2* mutant compared to Col-0 wild type under As (III) stress (Fig. 3g-j), which further supports that increased biosynthesis of GSH results in arsenite resistance of *siz1* mutants.

**Fig. 3:**
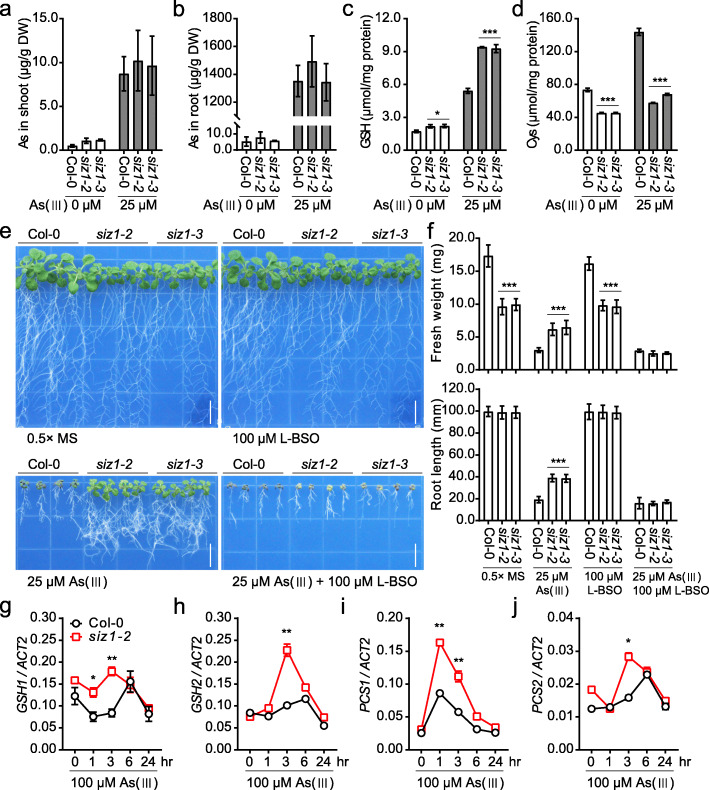
Arsenite-resistant phenotype of *siz1* mutants is suppressed by the inhibition of GSH accumulation. **a**, **b** Arsenic contents measured by ICP-MS analysis in shoot (**a**) and root (**b**) of 7-day-old Col-0, *siz1–2*, and *siz1–3* seedlings after As (III) treatment for 4 days. **c**, **d** The contents of GSH (**c**) and cysteine (**d**) in 7-day-old Col-0, *siz1–2*, and *siz1–3* seedlings after As (III) treatment for 4 days. **e** The effect of L-BSO on arsenite-resistance of *siz1* mutants. Five-day-old seedlings grown on 0.5× MS medium were transferred to the medium supplemented with 25 μM As (III), 100 μM L-BSO, or combination of 25 μM As (III) and 100 μM L-BSO for 12 days. Bar, 1 cm. (**f**) Fresh weight and root length from the seedlings shown in (**e**). Values are means ± SD of three replicates, each replicate containing 9 plants per genotype. *** *P* < 0.001, Student’s *t* test. **g** - **j** The transcript abundance of *GSH1* (**g**), *GSH2* (**h**), *PCS1* (**i**), and *PCS2* (**j**) determined by qRT-PCR analysis in Col-0 and *siz1–2* seedlings after 100 μM As (III) treatment for the indicated time. The *ACT2* was used as an internal control. Values are means ± SD (*n* = 3). * *P* < 0.05, ** *P* < 0.01, Student’s *t* test

Increased expression of the GSH biosynthetic genes in *siz1* mutant suggested a transcriptional regulation conferring SIZ1-mediated arsenite response. The transcription factor PHR1 is a master regulator for phosphate uptake and implicated in arsenic stress response (Navarro et al., 2021). PHR1 was also shown to be a SUMOylation target of SIZ1 (Miura et al., 2005). We therefore tested whether *PHR1* gene is involved in GSH biosynthetic regulation and arsenite tolerance in *siz1* mutant. The expression levels of *GSH1*, *GSH2*, *PCS1* and *PCS2* were comparable between the *phr1* mutant and Col-0 wild type (Fig. 4a-d), indicating that SIZ1-mediated arsenite response is unlikely through the function of PHR1. The dwarf-like phenotype of *siz1* mutant is caused by increased accumulation of salicylic acid (SA), which can be rescued by the expression of *nahG*, a bacterial salicylate hydroxylase that catabolizes SA (Miura et al., 2010). We tested whether SA accumulation is associated with arsenite response in *siz1* mutant by using the *siz1* mutant expressing *nahG*. The *siz1–2 nahG* showed a s response to As (III) similar to *siz1–2* mutant (Fig. 4e and f), suggesting that *SIZ1* modulates arsenite response via an SA-independent pathway in Arabidopsis. Since SIZ1 activates COP1 (CONSTITUTIVE PHOTOMORPHOGENIC 1), an ubiquitin E3 ligase promoting the degradation of the bZIP transcription factor HY5 (ELONGATED HYPOCOTYL 5), and HY5 is a central positive regulator in sulfur assimilation that provides the thiol group for GSH biosynthesis (Lee et al., 2011; Lin et al., 2016), we speculate that the transcription factor HY5 may be involved in SIZ1-mediated gene regulation in GSH biosynthesis. However, this requires further experimental validation in the future.

**Fig. 4:**
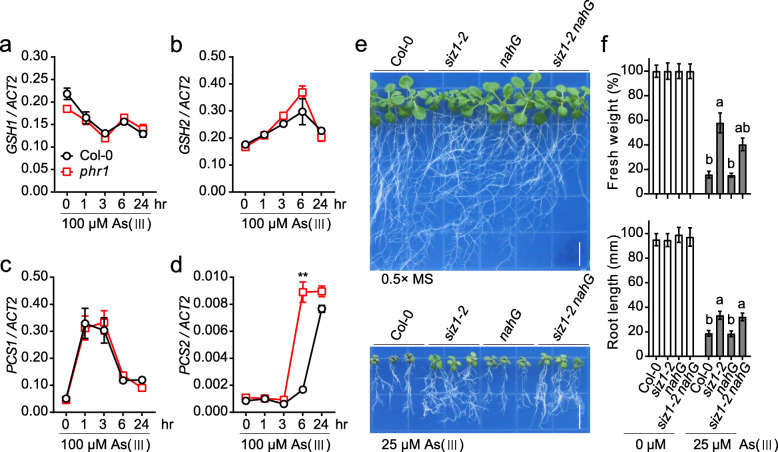
The effect of *phr1* mutation and SA accumulation on arsenite resistance of *siz1* mutants. **a** - **d** The transcript levels of *GSH1* (**a**), *GSH2* (**b**), *PCS1* (**c**), and *PCS2* (**d**) determined by qRT-PCR analysis in Col-0 and *phr1* seedlings after 100 μM As (III) treatment for the indicated time. The *ACT2* was used as an internal control. Values are means ± SD (*n* = 3). ** *P* < 0.01, Student’s *t* test. **e** Arsenite resistance assay of Col-0, *siz1–2*, *nahG*, and *siz1–2 nahG*. The seedlings grown on 0.5× MS medium were transferred to the medium containing 25 μM As (III) for 12 days. Bar, 1 cm. **f** The fresh weight and root length measured from the seedlings shown in (**e**). Values are means ± SD of three replicates, each replicate containing 9 plants per genotype. The letter a and b above columns indicate significant difference (*P* < 0.05, Student’s *t* test) among the values

In conclusion, we identified a novel function of SIZ1 in modulating arsenite response in Arabidopsis. Overexpression of the dominant-negative *SCE1*^*C94S*^ mimicking the *siz1* mutant suggests that protein SUMOylation negatively regulates arsenite resistance in Arabidopsis. Our results reveal that SIZ1-mediated SUMOylation modulates arsenite response through the control of GSH biosynthetic genes and thus the accumulation of GSH and cellular detoxification. Rapid industrialization and urbanization have accelerated arsenic pollution in agricultural land and water which adversely affects crop production and human health (Zhao et al., 2010). Our findings provide important genetic insights into plant adaption to heavy metal and metalloid stress and a possible target for gene editing to improve arsenite resistance in crops.

## Materials and methods

### Plant materials and growth conditions

In this study, all the Arabidopsis (*Arabidopsis thaliana*) genetic materials are in Columbia-0 background. The T-DNA insertion mutants of *SIZ1*, SALK_065397 and SALK_034008, were obtained from the Arabidopsis Biological Resource Center (ABRC). The *phr1*, *nahG*, *siz1–2 nahG*, transgenic lines had been reported in our previous study (Dong et al., 2019; Kong et al., 2020). After surface-sterilization and stratification at 4 °C for 48 h, the seeds were sown on 0.5× Murashige and Skoog medium (pH 5.8) containing 1% (w/v) sucrose and 0.6% (w/v) agar and grown in a growth room at 22 °C with 16 h light / 8 h dark condition. To generate the complementation lines, a 2 kbp promoter of *SIZ1* was amplified and cloned into the upstream of 3×HA in pCambia1305 vector. The CDS of *SIZ1* was then cloned into the downstream of 3×HA to generate the *SIZ1pro:3*×*HA-SIZ1* construct. The construct was introduced into *siz1–2* plants by *Agrobacterium tumefaciens* GV3101 using the floral dip method. The homozygous T4 plants were used for the analyses.

### Phenotype assays

For As (III) and As(V) resistance assay, five-day-old seedlings grow on 0.5× MS medium were transferred to 0.5× MS medium containing As (III), As(V), and/or L-BSO. After growth for 12 days, the plates were photographed and the fresh weight and root length were measured. The experiments were performed three times, each containing nine plants per genotype.

### Gene expression analysis

12-d-old seedlings were treated with exogenous 100 μM As (III) for indicated times. Total RNA was extracted using TRIzol reagent (Invitrogen). Reverse transcription was carried out using One-Step gDNA Removal and cDNA Synthesis Supermix (TransGen Biotech), followed by quantitative PCR on a CFX96™ Real-Time system (*BIO-RAD*) with ChamQ SYBR qPCR Master Mix (Vazyme Biotech co., ltd). Each analysis included three biological replicates. *ACT2* was used as an internal control.

### Immunoblot analysis

Immunoblot analysis was conducted as described previously (Hong et al., 2020). In brief, 10-day-old seedlings were collected and total protein was extracted using the extraction buffer (50 mM Tris-HCl, pH 8.0; 400 mM NaCl; 0.5% (v/v) Nonidet P-40; 10% (v/v) glycerol; 1 mM EDTA; 1 mM dithiothreitol; and 1 mM phenylmethylsulfonyl fluoride). Total proteins were separated in a 10% SDS-PAGE gel and electroblotted to NC membrane (Millipore), and the abundances of 3×HA-SIZ1 were then determined using anti-HA antibody (Roche). For profiling of SUMO1-cojugated proteins after As (III) treatment, 10-day-old seedlings were collected and subjected to 100 μM As (III) for indicated hours. Total protein was extracted and used to determine SUMOylation profiles using an anti-SUMO1 antibody (ab5316, Abcam).

### Elemental and metabolites analysis

Ten-day-old seedlings grow on 0.5× MS medium plates were transferred to 0.5× MS medium with or without 25 μM As (III) for additional 4 days. Arsenic contents were measured using inductively coupled plasma mass spectrometry (ICP-MS) as described in previous studies (Chao et al., 2014; Wang et al., 2020). Briefly, the shoots and roots of the seedlings were sampled separately and diluted to 10.0 mL with deionized water after digesting with 0.90 mL nitric acid. Elemental analysis was performed with an ICP-MS (NexION 350D; PerkinElmer) coupled to an Apex desolation system and an SC-4 DX auto sampler (Elemental Scientific Inc., Omaha, NE, US). The content of cysteine and GSH were measured by using cysteine and glutathione assay kits (NJJCBIO, China) following the manufacturer’s instructions.

#### Accession numbers

Sequence data from this article could be found on the website of Arabidopsis Information Resource (www.arabidopsis.org) under the following accession numbers: *GSH1*, AT4G23100; *GSH2*, AT5G27380; *PCS1*, AT5G44070; *PCS2*, AT1G03980; *PHR1*, AT4G28610; *SCE1*, AT3G57870; *SIZ1*, AT5G60410; *ACT2*, AT3G18780.

## Data Availability

All data generated or analyzed during this study are included in this published article.

## Abbreviations

COP1: CONSTITUTIVE PHOTOMORPHOGENIC 1
GSH1: *γ*-glutamate-cysteine ligase
GSH2: glutathione synthetase 2
HY5: ELONGATED HYPOCOTYL 5
L-BSO: L-buthionine sulfoximine
MS: Murashige and Skoog
PCS1: phytochelation synthase 1
PCS2: phytochelation synthase 2
PHR1: phosphate starvation response 1
qRT-PCR: quantitative reverse transcription-PCR
SA: salicylic acid
SCE1: SUMO conjunction enzyme 1
SIZ1: SAP and MIZ1 domain-containing ligase 1
SUMO: small ubiquitin-like modifier
T-DNA: transfer DNA.
